# Dealing with the difficult student in emergency medicine

**DOI:** 10.1186/1865-1380-4-39

**Published:** 2011-06-29

**Authors:** Sarah E Ronan-Bentle, Jennifer Avegno, Cullen B Hegarty, David E Manthey

**Affiliations:** 1 University of Cincinnati College of Medicine, Department of Emergency Medicine, Cincinnati, Ohio, USA; 2 LSUHSC Section of Emergency Medicine, New Orleans, LA, USA; 3 Department of Emergency Medicine, Regions Hospital, University of Minnesota, MN, USA; 4 Department of Emergency Medicine, Wake Forest University School of Medicine, 5th Floor Watlington Hall, Medical Center Boulevard, Winston-Salem, NC 27157, USA

## Abstract

Dealing with a student who is perceived as difficult to work with or teach is inevitable in any academic physician's career. This paper will outline the basic categories of these difficulties pertinent to Emergency Medicine rotations in order to facilitate appropriate identification of problems. Strategies for evaluation and reporting of the difficult student are presented. Remediation, based on the type of difficulty, is addressed. Timeliness of reporting, evaluation, and feedback are invaluable to allow for appropriate assessment of the outcome of the remediation plan.

## Introduction

As Emergency Medicine (EM) has grown in popularity as a specialty, so has the number of medical students who participate in an EM clerkship during their school years. At present, over 160 distinct student rotations are offered in EM at US medical schools (informal review by David Manthey, 2010). Medical student educators in every specialty occasionally encounter students who have difficulties during their clerkship. Although there is little in the EM literature that describes the prevalence of the difficult learner interaction, reports from other specialties suggest that up to 15% of learners are identified as "struggling" or "problem" learners during their clinical rotation [1]. Although disagreement often exists as to what constitutes a difficult or unsuccessful learner experience, identification of problem learners is important. Unprepared or unprofessional students may become practicing physicians with inadequate knowledge of acute medical conditions and provide inappropriate care. Furthermore, these learners can go on to have significant negative impact on the medical and larger community, as it has been shown that unprofessional student behavior often correlates with future problems and formal disciplinary action [2].

Any EM physician who mentors, teaches and/or trains medical students should understand and identify difficult learner interactions, and be prepared to deal with these situations appropriately. This paper describes the types of difficult learner interactions in EM, strategies for proper documentation and planning, and remediation procedures.

### Identifying the difficult learner interaction

**Figure 1:**
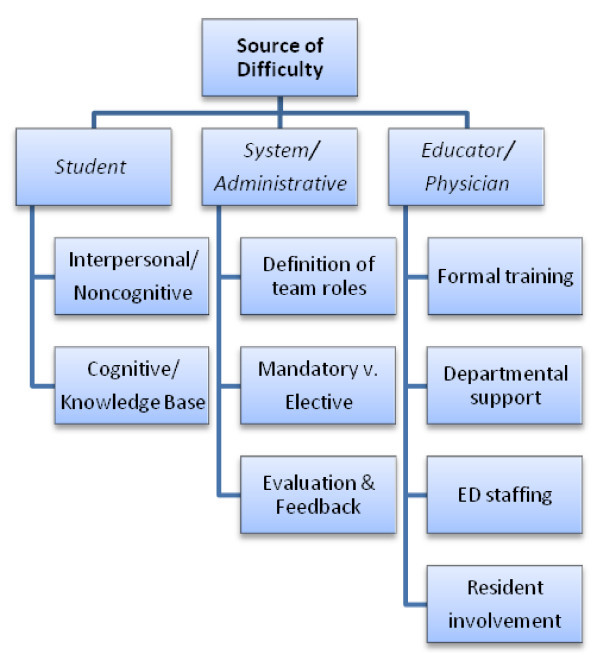
**Summary of sources of difficulties that learners may experience during an EM rotation**.

The Emergency Department (ED) is a challenging place to train and presents a unique set of conditions that may contribute to a difficult learning experience. Problems that occur on an EM rotation may be caused by one or more factors: learner characteristics, system/administrative constraints and educator issues (Figure 1).

#### Learner characteristics

Student-centered problems with the EM rotation can be divided into two categories: cognitive and non-cognitive/interpersonal. Cognitive or knowledge base difficulties are often easily identified both on-shift and in the didactic setting. Students may be unprepared for one or more aspects of the clerkship (patient care, academic work, formal evaluation methods). Such deficits may be due to poor preparation for the rotation, underdeveloped critical thinking skills, insufficient attention to studies, or a learning disability. The unknowledgeable learner may show significant gaps in basic clinical knowledge, diagnosis, and management of patients, and may perform poorly at the bedside and on objective testing. Although these learners may be frustrating to supervising physicians, their issues are often the most straightforward to identify and remediate.

Non-cognitive or interpersonal difficulties are often more challenging. Although many educators are uncomfortable with addressing issues of professionalism, it is a core tenet of both undergraduate and graduate medical education. Included in the standard core competencies set forth by the Accreditation Council for Graduate Medical Education (ACGME) is the expectation that learners should demonstrate basic aspects of professionalism: integrity, respect for others, responsiveness and sensitivity to patients, and accountability [3]. Difficulty with any of these tenets demonstrated by learners may be due to differing belief systems, lack of awareness, or conflicting expectations of the EM rotation experience. If a problem with a learner's professionalism is identified, the possibilities of personal stressors or substance abuse should always be considered. After inquiring with the learner and eliminating these possibilities from consideration, other explanations can be explored.

Different learning styles may contribute to a student being thought of as problematic. Students who learn best in a traditional academic classroom setting may be overwhelmed by the learn-as-you-go, on-shift teaching model of many EDs. Learning disabilities should also be considered in those students who appear to have difficulty with basic knowledge or processing skills.

Another type of learner who might demonstrate symptoms of difficulty deserves special mention: the gifted learner [4]. These are learners who perform at the top on standardized tests or IQ tests. They often have exceptional creativity or notable achievements at young age [5]. Gifted learners in adulthood can achieve significant professional and social success if they learn how best to utilize their skills in societal frameworks. If gifted learners do not develop necessary coping skills their exceptionality can actually impede success in many areas of their lives, including careers.

#### System/administrative constraints

The setup and structure of an EM rotation may contribute to a difficult learner experience. Although the degree of student autonomy varies across rotations, it may be significantly different from what the learner has previously experienced in a clinical setting. Learners with limited team roles that are not clearly explained or enforced may be frustrated and lose interest. Conversely, students who are unaccustomed to significant autonomy in patient care may be unwilling to accept increased demands and unsure how or if to ask for help. Students participating in a mandatory EM rotation who do not plan to pursue it as a specialty might be more easily thought of as problematic because they may not show the same enthusiasm or commitment to the work as an EM-bound student. Evidence suggests that EM-interested learners need less remediation and are less frequently identified as having problems with effort or motivation [6].

Structure the EM clerkship to minimize systemic problems. Clearly stating expected goals, objectives, and rotation details should be done on the first day of the rotation. These expectations should ideally be given verbally, and then reinforced in writing (handouts or online) so that the student may easily reference them throughout the rotation. The student's role in the ED and as part of the health care team should be clearly defined and demonstrated, including a thorough tour of all patient care areas and explanation of ED-specific policies and methods of documentation. Expectations of professionalism should also be clearly stated, with examples provided if possible.

Methods of evaluation, feedback, and grading should be discussed at the rotation's start and available for review at any time by the student. Both direct (written and oral exams, simulation or skills lab testing) and indirect (assessment of on-shift performance) means of grading should be explained. Structuring clerkship evaluation forms to include descriptive feedback may contribute to increased detection of professionalism deficiencies when compared to a standard checklist [7]. Students should also be aware of their opportunities to evaluate faculty and resident physicians, as well as the clerkship experience itself.

#### Educator issues

The ED rotator often encounters a wide variety of supervisors in the ED-academic and clinical staff, residents, and interns. Supervising physicians may not be formally trained in teaching, evaluation and feedback methods. This may lead to varying degrees of comfort with and methods of assessing student performance.

Each EM department has differing levels of support for educational activities; if supervising physicians do not feel adequately compensated and encouraged to participate in teaching, they may be less understanding of learner needs and styles. Similarly, if an ED is inadequately staffed to support educational pursuits, busy physicians may view EM-naive students as a hindrance rather than an asset, and may be more likely to label them as "difficult." Residents-who generally derive no direct compensation from additional academic activities-may feel as if the demands of student learning are a challenge to their own training opportunities.

The difficult learner interaction in the ED may be multi-factorial, with elements of student, system and educator all contributing to the problem. Attempting to pinpoint the source(s) of difficulty is critical to proceeding with feedback and remediation.

### Collection of objective data on the difficult learner interaction

During the EM clerkship it is important to collect feedback from multiple sources to identify the learner needing remediation-in the areas of knowledge, skills or attitudes. Testing is one way to identify learners with medical knowledge (or test-taking) issues. For many EM clerkships, a final exam is taken at the end of the rotation, making it difficult to have time to remediate a potential knowledge deficit until the rotation is complete. One suggestion is to have a pre-test for the clerkship and/or weekly quizzes to identify these students earlier. Assessment of student performance in the ED is a way to identify the full spectrum of problems a student may have. Daily shift feedback cards along with formative feedback directly to the student is a great way to assess performance on each shift, to document that performance and to give the student some real time feedback, allowing them the ability to improve each day on the rotation. Daily shift cards coupled with a mid-rotation feedback session for each student are one way to truly identify students needing remediation and establish a plan of action during their clerkship. The alternative of collecting feedback and only reviewing it at the end of the rotation may be less labor intensive, but will miss the window of opportunity to remediate students during their clerkship. Early detection is key.

One additional source of feedback to identify students' attitudes and professionalism is to seek input from the clerkship coordinator and all of the non-physician staff with whom the students interact during the clerkship-clerks, techs, nurses, etc. Some students that have professionalism issues can 'hold it together' in front of staff and residents, but may show their true colors during their interactions with other members of the ED team and rotation. Setting the expectation for the learners and evaluators prior to the start of the rotation that our job is to improve each individual medical student, and not to just provide them with an 'experience' in Emergency Medicine, will open the door for the possibility of providing real formative feedback to the student.

Even if you set up your rotation in a well planned out format to provide students with the best opportunity to learn and improve, there are certain barriers to be aware of that may interfere with your ability to truly identify and remediate a student in need: poor documentation, generic and often positive verbal feedback, lack of direct observation of performance, poor understanding of resources available to assist, and fear of 'negative' consequences for the evaluator and student.

### Documenting the difficult learner experience

Once a difficult learner has been identified and data gathered, the learner must be given feedback and become engaged in the process of managing the difficulty so as to improve the rotation experience for the learner and increase his/her likelihood of success. Integral to this process is documentation of the issues associated with the difficult learner.

One approach in developing an action plan is the SOAP method, adapted by Langlois and Thach for implementation in the outpatient Family Medicine setting [8]. Such a process has not been described or validated in the ED setting, but does provide a valuable framework for educators. The SOAP method allows for gathering data, making objective assessments, developing a differential diagnosis and plan of action, summarized in Table 1.

**Table 1:** SOAP method allows for gathering data, making objective assessments, developing a differential diagnosis and plan of action

	Definition	Examples
**Subjective**	*Describe the chief complaint of the behavior*	"Student is repeatedly late to shift" "Student cannot provide appropriate differential diagnoses for patient presentations"
**Objective**	*List specific instances of behavior*	"On night shift of 4/15, student arrived 45 min after scheduled start of shift" "After interviewing a patient with altered mental status, student's only diagnosis was intoxication"
**Assessment**	*Differential diagnosis of the difficulty*	Lateness = professionalism, attitudinal Inadequate knowledge base = cognitive
**Plan**	*Detailed course of action, with learner input*	"Student will arrive 10 min early for each shift and must have shift card signed upon arrival" "Student will read core chapters on selected EM topics and be able to list differential diagnoses for several basic EM patient presentations"

#### SOAP method

##### Subjective

In the subjective component, the educator describes the chief complaint such as "lazy, overbearing, chronically late, etc." How is the difficulty surfacing? Is this noted by more than one person or in multiple interactions? It is important to gather data from those in the clinical environment, including residents and staff who may have encountered the difficulty as well as office personnel who can recognize interpersonal difficulties. These data describe the "symptoms" of the chief complaint.

##### Objective

This is simply a list of the specific instances of behavior that illustrate the chief complaint and symptoms. Again, obtaining this data from multiple sources who have had experience with the learner proves helpful in illustrating the difficulty. It is important that this information is free of subjective interpretation of the incident.

##### Assessment

This involves formulation of a differential diagnosis of the difficulty based on the subjective and objective information. Analyzing the objective behaviors and the symptoms of the difficulty and categorizing them in the following general areas can help flush out the differential diagnosis of the difficulty. These categories include: Cognitive and Non-cognitive, Professionalism, and Attitudinal [8].

##### Plan

The plan requires input from the learner. As such, a specific meeting to discuss rotation feedback is helpful. The learner's involvement is important for buy-in and planning interventions for the difficulty. Setting expectations for following up on the proposed intervention to determine effectiveness should also occur at this meeting. This planning stage also potentially allows for gathering information from the institution regarding the learner's performance on other rotations and whether a pattern of difficulty is isolated to the ED rotation or more pervasive through other rotations.

### Feedback for the difficult learner

Giving detailed specific feedback to the learner is the foundation for formulation of the plan and for ultimately effecting change in behavior. Multiple models for delivering feedback to learners have been described [9-11]. A model developed for the outpatient Family Practice office setting involves four steps with the acronym TIPS [9].

#### TIPS feedback

First, Type and specify the ineffective behaviors. This requires giving specific examples of the difficult behavior and details about the difficulty. The next step is to Identify the category of difficulty experienced by the learner. This is similar to the assessment as developed in the SOAP method above.

The educator summarizes this assessment for the learner. In the third step, described as "perception versus reality feedback," the educator describes the difficulty as his/her Perception of the behavior(s) to the learner [9]. It is important to allow the learner to express his/her thoughts about the difficulty, recognizing that the learner's view may be very different.

With the final step in the feedback process, the learner and educator together develop Strategies for treatment and follow-up. These strategies may be specific to the rotation; however it is important to assist the learner to understand how these strategies may be applicable to future rotations or other aspects of training.

### Remediating the difficult learner

In medical school and for student clerkships, remediation is often thought of as the process of improving and reassessing a specific area of focus for the medical student. Remediation is defined by the Federation of State Medical Boards to have three components. First, deficiencies in performance are identified through an assessment process; second, an attempt is made to provide remedial education; and finally, the individual is reassessed in the area of his or her deficiency [12]. Depending on when the deficiency is identified along with the severity of the issue, the specific remediation plan may be able to be created and completed during the duration of their EM clerkship. If this is not successful or possible then the medical school process should be activated. This process will vary from school to school, and it is recommended to be familiar with the resources and policies of your specific institution.

The 2009 Academic Medicine article 'Remediation of the Deficiencies of Physicians across the Continuum from Medical School to Practice: A Thematic Review of the Literature' by Hauer et al. provides an outstanding review of the literature on deficit remediation at the undergraduate, graduate and continuing medical education levels along with research from the learning sciences [12]. Most of the literature on this topic comes from case reports, and there is no single 'best practice' or 'evidence-based' approach that has been well studied. Taking the best parts of the current remediation literature combined with concepts and research from the learning sciences, the Hauer group proposes four essential elements of a successful remediation program: (1) initial assessment (or screening) using multiple assessment tools to identify deficiencies, (2) diagnosis of problems and development of an individualized learning plan, (3) provision of instruction that includes deliberate practice, feedback and reflection, and (4) reassessment and certification of competence [12]. These elements can be applied to the specifics of an EM clerkship to establish a remediation plan.

During remediation, provide instruction that includes deliberate practice, feedback and reflection. It will be necessary to tailor the program to the area of focus-knowledge deficits can be dealt with using focused reading and referrals to a university learning specialist, skill deficits may need practice sessions with simulation or direct observation, and professionalism issues may need mentorship and a more behavioral approach. Whatever the remediation activities may be, they should allow students the chance to have practice in that focus area immediately followed by specific verbal and written feedback [12]. The verbal, formative feedback will allow the student to get a sense of how they are progressing with their plan, and the written feedback will be very valuable for the clerkship director to have to support their final decision regarding the success or failure of the remediation plan.

#### Strategies for remediation

Specific strategies for treatment and follow-up differ significantly depending on the category of difficulty. A difficult learner with a cognitive deficit will require educational tools. A knowledge base deficit can be improved with additional reading or discussion on a specific topic. When the area of cognitive difficulty is procedural competence, high-or low-fidelity simulation, video review or other additional practices are effective components of a remediation plan.

Learners with professional or interpersonal difficulties often require more time and effort due to association with a person's behavioral pattern. Therefore, other experts within the learner's training should be involved with assessment of the difficulty as well as devising and implementing a plan for treatment. This category of difficulty often is recurrent and pervasive. Strategies may include videotaping, role playing or simulation to provide additional coaching in interpersonal skills. Specific counseling with a psychiatrist or other trained professional may be necessary.

Strategies for improving difficulties for the gifted learner include incorporating specific learning opportunities that challenge the learner while ensuring that the learner fulfills the rotation objectives and requirements [4]. Collaboration with the learner is particularly valuable in forming a strategy for improvement.

#### Re-evaluation

As students progress through their remediation plan it is important to get final information or 'retesting' to decide if the agreed upon expectations of performance have been achieved and competence can be certified [12]. Depending on the area of focus for the student, this final assessment of performance will need to be tailored. Examples include a written final exam to reassess medical knowledge, direct observation of skill or performance, or a final meeting to debrief experiences with professionalism and lessons learned to reassess behavioral issues.

During the entire remediation process from onset until completion, use the expertise available to your clerkship or site or medical school to help the student to the best of your ability. Staff at your site or on your clerkship may be expert instructors, and can mentor and instruct the student in a focus area. Your medical school will likely have examples of what other clerkships have done to remediate a particular issue, and also have experts such as learning specialists, master tutors, and professionalism and humanism leaders. In addition to having one point person from the clerkship to rely on for advice and mentorship during this likely difficult time for the student, it is wise to have them notify their formal medical school advisor and any other mentors they may have in order to have an adequate support system in place as they travel through the remediation plan. For many of these students this may be the first time in a long while that they have 'failed' to some degree, and there is also the threat of delaying completion of or failing out of medical school, causing a great degree of stress and concern for them.

As noted earlier, communication with the student and documentation of the issues are important in the early part of the remediation process while informing the student that there is an issue and coming up with the remediation plan. Additional file 1 is a student remediation template with some descriptors listed next to each category to assist with the formulation and documentation of a remediation plan. This communication and documentation theme continues on during and after the remediation plan is complete; keep the student well informed during the remediation process of how he/she is progressing with continuous feedback and document these details. Once the remediation process is complete, have a final face-to-face meeting with the student to inform them of their final assessment and grade for the clerkship, add this information to the documentation about the student, and then submit a final written report to the medical school for their files. All written documentation should be kept on file as long as possible in case it is needed later [13].

## Conclusion

The difficult student interaction requires early and frequent assessments of behavior by all parties involved. Students should be provided with rules and expectations of the rotation as well as criteria for evaluation. Concrete examples of problems need to be gathered and then discussed with the student. The student should be allowed an opportunity for self-assessment and be involved with the plan of remediation. If possible, remediation should occur during the rotation with adequate time and effort given to re-assessment. If the issue is recurrent or deemed too large to address during the clerkship, appropriate medical school support should be garnered. Documentation of the entire process is essential.

## Competing interests

The manuscript, as submitted or its essence in another version, is not under consideration for publication elsewhere, and will not be published elsewhere while under consideration by International Journal of Emergency Medicine. The authors have no declarations of competing interest, financial or otherwise, to report.

## Authors' contributions

All authors have made substantive contributions to the study, and there are no copyright constraints. All authors read and approved the final manuscript.

## Supplementary Material

Additional file 1 **Student remediation template**. Click here for file
