# Supplementary material for: Dealing with the difficult student in emergency medicine
Source: Int J Emerg Med. 2011 Jun 29;4:39. doi: 10.1186/1865-1380-4-39 (PMC3141387; doi:10.1186/1865-1380-4-39)
Supplement: Additional file 1 — Student remediation template. [file 1865-1380-4-39-S1.DOC]

ATTACHMENT 1. **STUDENT REMEDIATION TEMPLATE: EMERGENCY MEDICINE**

Student:

Clerkship and site:

Date:

Subjective: (describes the chief complaint such as “lazy, overbearing, chronically late, etc.”)

Objective: (list of specific instances of the behavior which illustrate the chief complaint—be specific with detailed information, and use multiple sources if possible)

Assessment: (formulation of a differential diagnosis of the difficulty based on the subjective and objective information)

Plan: (individualized learning plan that includes:

-provision of instruction that includes deliberate practice, feedback, and reflection

-inclusion of the student's faculty advisor and/or mentors for support and assistance

-reassessment and certification of competence)

**remember to document the process from start to completion**
